# Tropical Abscess of the Liver

**Published:** 1909-08-07

**Authors:** 


### SPECIAL ARTICLE.

### TROPICAL ABSCESS OF THE LIVER.

Tropical abscess of the liver is not less interesting from the point of view of its pathology, than it is from the point of view of its successful treatment, and particularly at the present time when a new method of treatment, founded on a sound, pathological basis, is slowly but surely making headway.

The causation of liver abscess is pretty generally held to be the activity of that particular variety of *amœba coli* which is so constantly found to be associated with the form of dysentery met with in the Tropics. This does not necessarily imply that an individual must have had an acute attack of dysentery previous to acquiring his hepatic condition, but it is probable that in nearly every case there must have been *some* dysentery; enough, at all events, to give the amœba a *raison d'être* for its continued presence in the intestine. How the amœbæ reach the liver is not so certain, whether *via* the lymphatics or the portal blood-stream. In all probability it is by the latter, and once having reached the finer ramifications of the portal vein, they settle there and produce inflammation of the liver parenchyma.

The onset of liver abscess is often so insidious that it is sometimes said that it may arise in very much the same way as the "cold" abscesses of tuberculous origin, without any acute inflammation of the liver having preceded its formation. It is not impossible that in these cases the preliminary hepatitis has been confined to the more central parts of the liver, and that consequently—just as in the case of a central pneumonia—the symptoms are atypical and misleading, owing to the fact that the serous coverings of the organ are not involved in the inflammation. The subsequent development of an abscess in a case of this sort may then come as a complete surprise to both doctor and patient, localising symptoms never having been definite enough to attract attention to the liver.

From a clinical point of view, these cases vary so much that it would be possible to arrange them in a large number of classes, each distinguished from its fellows by the presence or absence, or the greater prominence of some particular symptom. But such a division would be quite artificial, for while there are, on the one hand, the very acute fulminating cases where the onset is characterised by acute pain and tenderness over the liver, high temperature, quick pulse, vomiting and rigidity of abdominal walls; and, on the other hand, slow insidious cases—characterised by no acute symptoms, and ending, perhaps, in the formation of an enormous abscess for which the patient seeks relief owing to "shortness of breath!" There is between these two extremes every type of case. In fact, it is probable that only in the case of appendicitis have we a disease so protean in its manifestations and symptomatology as is liver abscess. Sometimes the abscesses are multiple, but fortunately they are usually single, or have become so before surgical intervention takes place. It is probable that, if many abscesses are present another element of infection is present, and that portal pyæmia of staphylococcal or coli origin has occurred.

In considering some of the clinical aspects of the disease, it is naturally the more chronic cases which will particularly interest those in England, for the fulminating type of abscess is not often met with outside the tropics. They may be very easy of diagnosis, but, on the other hand, they are often very difficult. The patients turn up sometimes after a residence in England of many months or even years, complaining of loss of appetite, emaciation, want of energy and general malaise; and that is all. There is not a word about the liver! There may be a muddy brown complexion which to the experienced is a very valuable indication, and there may be a history of profuse sweats, and perhaps an evening rise of temperature to 99.5 or 100.

When examination of the hepatic region is carried out, the typical dome-shaped area of dulness so frequently described is often absent; perhaps the lower margin of the liver is only just palpable, and the dulness extends but one rib higher than normal. What is one to do in such a case? Is it a case of abscess, or is it merely chronic hepatitis of malarial or alcoholic origin? It is here that skiagraphy has proved so useful. An *x*-ray photograph will not reveal an abscess by any difference in the density of the shadow, but it will at once show up any abnormal bulging, or other irregularity, in the outline of the liver. And it must be obvious that a considerable localised bulging might be possible in various directions without giving rise to any material alteration in the liver dulness, and without being apparent to ordinary palpation.

The last step in diagnosis is to thrust an exploring needle into the liver and actually demonstrate the presence of pus. But it is necessary to remember several important points in connection with this relatively simple procedure. In the first place it is well never to delay making an exploratory puncture, for even if no pus is found no harm is done. But when the puncture is made the patient must be anæsthetised and the surgeon prepared, so that in case pus is found, it may at once be further dealt with according to the method it has been decided to use. And finally, in using an exploring needle in the liver, just as in the brain, the needle must never be moved laterally, but must be taken out and re-inserted if the first exploration is unsuccessful.

The actual treatment of abscess has for many years been on the great surgical principle that all abscesses, whatever their nature or situation, should be freely opened and drained. And this method of treatment often gives good results; but, on the other hand, it frequently happens that the result is not so happy. In the first place the patients are often so pulled down as hardly to be in a condition to stand the really extensive operation involved in resecting portions of one or more ribs, and opening the liver; secondly, liver tissue is unsatisfactory material to cut into; and, thirdly, the pus in these abscesses is of such a character that no known dressing will absorb it, with the result that it runs down the chest wall under the dressings, and most of it finds its way into the bed.

The pus of a liver abscess is peculiar in many ways; thick, viscid, and the colour of anchovy sauce, its very appearance is unlike anything else. It is peculiar also from the fact that, as regards ordinary organisms it is quite sterile both in films and cultures. If, however, a hanging drop preparation be examined on a warm stage, the amœba coli may be recognised, pushing out their pseudopodia.

It appears to be certain that the pus is the result of the activity of these protozoal organisms, and of them alone. Acting on this assumption Captain L. Rogers, of the I.M.S., conceived the possibility of destroying the amœba *in situ* by the injection of a fluid destructive to protozoa, and he suggested for this purpose a solution of quinine, which is so highly poisonous to other forms of protozoa, as, for instance, spermatozoa or the organisms of malaria.

His suggestions have been adopted and the method has been followed by brilliant results. The procedure is as follows:—The patient is prepared and anaesthetised in the usual way, and an exploring needle is thrust through the eighth or ninth interspace. A sudden loss of resistance will usually indicate when the abscess has been reached, but if this is not the case, the needle must be pushed in three or four inches, and then an aspirating bottle is attached and the needle slowly withdrawn. It is important to use a large needle in view of the thick and viscid consistency of the pus, and it is wise to use not too great a vacuum pressure in order to diminish hæmorrhage. When the pus has been struck it is slowly aspirated away until the abscess cavity appears to be empty, great care being taken that in the manipulation of the needle the point is not withdrawn from the cavity. This may easily happen, and there is no other indication of its occurrence than the cessation of the pus flow, which will probably be ascribed to the complete emptying of the abscess sac.

The next proceeding is to replace the pus by a quantity of 1 per cent. solution of bichloride of quinine; a tube and funnel are attached to the needle and the solution is allowed to gravitate in, with the funnel held about three feet above the level of the patient. Opinions differ as to the quantity of solution which is to be allowed to run in. Some hold that a quantity equal to the amount of pus removed should be used; but this is probably excessive, for there can be no doubt that the abscess cavity undergoes considerable diminution in size directly the pus is abstracted, and further the vacuum created will necessarily be followed by a certain amount of bleeding, which will tend to fill the cavity. It is probably sound practice to allow as much solution to enter as can be got to do so with a pressure of about two feet, and only to use greater pressure until an amount equivalent to half the pus abstracted has run in. The patient quickly recovers, and usually expresses himself as much relieved, but some pain in the upper part of the abdomen may be complained of for a few hours after the operation. This is in all probability due to the escape of a few drops of quinine solution into the peritoneum.

Almost immediately after the operation the temperature falls to normal, the colour rapidly improves and the leucocytosis drops several thousands, and in some cases the patient will get gradually but completely well. Often, however, the pus collects again, and if this is the case, on the third or fourth day the temperature will rise, the leucocyte count will increase and the pigmentation of the skin will return; the patient will at the same time lose his appetite and begin to have restless nights again. Aspiration and injection should now be carried out again without delay, and if necessary even a third or fourth time. On each occasion it will be found that the quantity of pus has diminished. Thus in one case, at the first operation there were 57 oz., at the second 30 oz., and at the third and last 20 oz., the patient then getting completely well.

It is probable that in the case of these very large abscesses one operation is never sufficient, and there may be other factors which are of importance in determining the ultimate fate of the case. For instance, in another case, after five aspirations, the quantity of pus withdrawn each time had diminished from 20 to 5 oz., but still it re-collected. The patient was therefore subjected to the ordinary operation, when it was found that the abscess was so superficial that the chest wall practically constituted its outer boundary.

As it is obvious that complete cure of this condition can only be brought about by collapse of the abscess cavity, the major operation was necessary here in order to allow the outer wall of the abscess to fall in. But even if these cases have ultimately to undergo a resection of rib, by the time this is carried out, the general state of the patient and the local condition of the abscess have been so improved by the injections, that recovery is rapid and uneventful.

It appears, therefore, that by this method of injection we have a means of treating tropical abscess of the liver of so simple and straightforward a nature that it can be utilised in such places and under such conditions as might render the older operation one of extreme difficulty and danger.

